# “Patient dignity can be ensured by providing adequate health care”: A phenomenological analysis on survival strategies of military nurses

**DOI:** 10.1016/j.heliyon.2024.e25893

**Published:** 2024-02-10

**Authors:** Mst. Rina Parvin, Priyanka Das Sharmi, Ibne Kayesh, Moustaq Karim Khan Rony

**Affiliations:** a Afns Major at Bangladesh Army, Combined Military Hospital, Dhaka, Bangladesh; b School of Medical Sciences, Shahjalal University of Science and Technology, Sylhet, Bangladesh; c Assistant Professor, International University of Business Agriculture and Technology, Dhaka, Bangladesh; d Institute of Social Welfare and Research, University of Dhaka, Dhaka, Bangladesh; e Master of Public Health, Bangladesh Open University, Dhaka, Bangladesh

**Keywords:** COVID-19, Healthcare, Military nurses, Patients, Pandemic, Survival strategies

## Abstract

**Background:**

The devastating COVID-19 outbreak has considerably influenced basic human healthcare needs. Due to healthcare organizational limitations, workload, and a shortage of healthcare professionals, particularly military nurses in developing countries, faced critical situations in dealing with COVID-19 patients. However, little is known about the survival strategies military nurses used while caring for coronavirus-infected patients.

**Aims:**

This study aimed to investigate the survival strategies that inspired military nurses to manage COVID-19 patients in Bangladesh.

**Methods:**

This study employed the phenomenology of practice framework developed by Max van Manen. Fourteen military nurses were purposefully selected to participate in this study. Semi-structured online interviews were conducted individually from January to February 2023 in three divisional (Dhaka, Chittagong, and Sylhet) COVID-19 dedicated military hospitals in Bangladesh. The study was reported using the COREQ checklist. Audio-video sessions of discussions were recorded, analyzed, and transcribed verbatim. Dataset analysis was performed using thematic analysis.

**Results:**

Eight themes were developed: (i) Nurses' self-strategies; (ii) colleagues' strategies; (iii) Nurse managers' strategies; (iv) Feelings about nursing ethical values; (v) Employers' strategies; (vi) Government strategies; (vii) Family members' strategies; (viii) Strategies of social people.

**Conclusions:**

The study's findings would inspire healthcare professionals to use various survival strategies when facing critical clinical situations. Additionally, this study encourages nurses to develop survival skills to avoid depression, fear, and anxiety and to learn how to deal with work-related stress situations.

## Highlights

• Nurses consciously avoided negativity and actively sought positive perspectives to alleviate stress and maintain an optimistic mindset.
• Nurses assisted their colleagues, creating a collaborative environment where knowledge and resources were shared.
• Nurses consistently embrace essential nursing ethical values, treating patients with respect, compassion, and empathy.
• Employers actively sought feedback from healthcare workers to improve the quality of services and promote open communication.
• Family members became a source of strength and motivation, reminding nurses of the importance of their work and the positive impact they were making.

## 1 Background

The COVID-19 pandemic had a profound and far-reaching impact on the world, unprecedentedly affecting global health, economies, social dynamics, and daily life. However, healthcare providers faced the challenges and devastation of this situation, with nurses playing a critical role in protecting individuals and communities during the pandemic [1]. They demonstrated unwavering dedication and commitment to patient care while implementing stringent infection control measures. Nurses were at the forefront of screening, testing, and treating COVID-19 patients, often putting themselves at risk of exposure [2]. Additionally, they educated patients and their families on preventive measures such as mask-wearing and social distancing. Their knowledge and compassion were crucial in halting the spread of the virus and providing quality nursing care to COVID-19 patients [3]. However, military nurses played a crucial role in caring for COVID-19 patients, particularly in developing countries with limited healthcare resources. Military nurses combined their medical expertise with the demands and discipline of the military environment [4]. Nevertheless, they faced numerous obstacles, such as limited resources, high patient volumes, and an increased risk of pathogen exposure [5].

Survival strategies are the specific actions, techniques, or approaches that individuals or groups utilize to overcome obstacles, adversities, or threats to ensure survival or well-being [6]. These strategies consist of physical, mental, emotional, and practical responses to navigate challenging situations, mitigate risks, and enhance survival [7]. Survival strategies can vary depending on the situation, including survival in the wilderness, during natural calamities, in times of crisis or conflict, or in challenging working environments within hospital settings [8]. These strategies frequently involve problem-solving, adaptation, resilience, resource management, collaboration, and utilizing available resources and support systems to endure and flourish under challenging circumstances [9].

Survival strategies encourage nurses to navigate the challenges of the clinical environment. By employing effective survival mechanisms, nurses can better manage stress, maintain their well-being, and deliver quality patient care [10]. Strategies such as seeking social support from colleagues, friends, and family; practicing self-care through exercise and relaxation techniques [11]; employing mindfulness and stress reduction techniques; seeking professional counseling when necessary; and maintaining a positive outlook all contribute to resilience and emotional well-being [12]. Effective time management, setting boundaries, and prioritizing tasks can also help nurses maintain a sense of control and balance amidst the demanding and sometimes overwhelming nature of their work [13].

In Bangladesh, military nurses routinely work in austere and hostile environments characterized by limited access to healthcare resources [14,15]. These dedicated healthcare professionals play a unique and indispensable role in the nation's defense, frequently encountering challenging situations that demand specialized coping strategies [16]. They are consistently exposed to traumatic events, which can profoundly affect their mental well-being [17]. Exploring their survival strategies can provide insights into effective resource allocation techniques that have the potential to be adapted for broader application within the healthcare sector, ultimately leading to enhanced resource utilization across the industry [18].

Research on survival strategies during the COVID-19 healthcare crisis has predominantly concentrated on civilian healthcare providers [19,20], leaving a noticeable gap in our understanding of frontline military nurses' unique circumstances. These nurses must navigate the dual demands of their military responsibilities and healthcare duties, making their experiences distinct [21]. Consequently, there is an imperative to specifically investigate the survival strategies embraced by frontline military nurses as they confront the challenges posed by caring for COVID-19 patients in developing nations. Moreover, gaining insights into how frontline military nurses cope with these distinctive challenges within such settings can offer invaluable contributions to our comprehension of global healthcare responses and the formulation of future preparedness plans for analogous crises. Thus, this study aimed to explore the survival strategies that inspire frontline military nurses while caring for COVID-19 patients in Bangladesh. Through a qualitative research approach that delves into their experiences, challenges, and coping mechanisms, this study provides meaningful insights that can aid healthcare professionals and individuals in navigating the intricacies of their roles within resource-constrained environments.

## 2 Methods

### 2.1 Design

The present study, grounded in Max van Manen's phenomenology of practice, adopted a phenomenological method to investigate the survival techniques that inspired military nurses in handling COVID-19-infected patients during the pandemic. Hermeneutic phenomenology focuses on how individuals perceive their unique experiences in the world [22]. This qualitative inquiry illustrates real-life experiences, events, and contexts, specifically within the nursing profession [23]. The method emphasizes capturing details and characterizing situations in human experiences. By employing a phenomenological approach, researchers can descriptively, appropriately, and dynamically reveal specific clinical circumstances. The themes identified in phenomenological analysis serve as the basis for understanding and characterizing the clinical experiences of nurses [24]. In this study, the authors thoroughly examined the data to extract codes, which were then utilized to generate themes that are relevant to the research purpose.

### 2.2 Participants

Participants for this study (Table 1) were recruited using purposive sampling from three divisional (Dhaka, Chittagong, and Sylhet) COVID-19 dedicated military hospitals in Bangladesh. Nurses who met the following criteria were included: (a) Possessing a minimum of two years of experience in dealing with COVID-19 patients: This criterion was established to ensure that participants had substantial practical experience in caring for COVID-19 patients. It helps in gathering insights from nurses who have encountered various scenarios and challenges related to COVID-19, thus contributing to a richer and more informed discussion. (b) Employed in a facility capable of admitting more than 100 COVID-19 patients: This criterion was set to focus on nurses working in larger healthcare facilities that handle a substantial volume of COVID-19 cases. These facilities often have different dynamics, resource availability, and challenges compared to smaller ones. By including nurses from such settings, the study can capture a broader range of experiences and perspectives. (c) Aged 25 or older: This criterion may have been established to ensure a certain level of maturity and life experience among participants. Older participants might have developed more well-rounded insights into their profession and coping mechanisms over time, which can contribute to a more comprehensive understanding of survival strategies during the pandemic. To accommodate participants' preferences, interviews were scheduled at their preferred times. Prior to obtaining their consent, participants were provided with information about the study's purpose, ethical approval, potential risks, and their rights as participants.

**Table 1:** Showing participant characteristics (N = 14).

**Code**	**Sex**	**Age (Years)**	**Marital status**	**Work experience (years)**	**Highest degree**	**Desingnation**	**Survival strategies**
101	F	33	Yes	10	Masters	Afns lieutenant	Self-strategy of nurses, Feelings of nursing ethical values, Employer strategies
102	F	26	No	3	Bsc	Afns lieutenant	Colleagues' strategies, Nurse managers strategies, Government strategies
103	F	38	Yes	15	Bsc	Afns Major	Feelings of nursing ethical values, Employer strategies, Social people's strategies
104	F	42	Yes	19	Bsc	Afns Major	Nurse managers strategies, Government strategies, Family members strategies
105	F	27	Yes	4	Bsc	Afns lieutenant	Self-strategy of nurses, Colleagues' strategies, Family members strategies
106	F	29	Yes	4	Masters	Afns Captain	Colleagues' strategies, Employer strategies
107	F	36.5	Yes	12.5	Masters	Afns Major	Self-strategy of nurses, Nurse managers strategies, Feelings of nursing ethical values
108	F	29.5	Yes	3.5	Masters	Afns Captain	Feelings of nursing ethical values, Government strategies, Social people's strategies, Employer strategies
109	F	31	Yes	7	Bsc	Afns Major	Self-strategy of nurses, Government strategies, Social people's strategies
110	F	26	No	2.5	Bsc	Afns lieutenant	Nurse managers strategies, Employer strategies, Family members strategies
111	F	35	Yes	12	Masters	Afns Major	Colleagues' strategies, Feelings of nursing ethical values, Social people's strategies
112	F	28	No	5	Bsc	Afns Captain	Nurse managers strategies, Government strategies
113	F	37	Yes	13	Bsc	Afns Major	Self-strategy of nurses, Employer strategies
114	F	32.5	Yes	9.5	Masters	Afns Captain	Colleagues' strategies, Family members strategies, Self-strategy of nurses

F = Female; Bsc = Bachelor of Science in Nursing; Afns = Arm forces nursing service.

The sample size for this study was determined through the data saturation process, which ensures that a sufficient number of effective survival strategies are explored. The process of data collection and analysis was repeated until no new survival strategies for dealing with the adverse conditions of COVID-19 emerged [25]. Three additional military nurses were interviewed to confirm the achievement of data saturation.

### 2.3 Data collection

Data collection for this study took place from January to February 2023, with the dataset gathered from dedicated COVID-19 military hospitals in Bangladesh. To recruit frontline military nurses caring for COVID-19 patients, the authors distributed a circular through various Bangladeshi Facebook and WhatsApp groups. Additionally, the authors obtained a list of military nurses' email addresses and phone numbers from the respective hospital authorities to extend invitations for online interviews.

Participants were selected using purposive sampling techniques, and the authors scheduled semi-structured online video interviews based on the participants' convenience. Out of the seventeen eligible participants, three declined to take part in the study; one cited a heavy workload, another had upcoming maternity leave plans, and the third expressed discomfort discussing their experiences with COVID-19 patients. Fourteen participants' interviews, conducted via the Zoom online platform, lasted approximately 40–45 min. It's worth noting that all interview sessions were conducted in the Bangla language. The interview guide consisted of five open-ended questions aimed at gaining insights into the survival strategies that inspired military nurses in coping with critical COVID-19 situations.

### 2.4 data analysis

The collected data, originally in the Bangla language, was translated into English. A data translation specialist was involved in this process to ensure the authenticity of the primary data and to maintain the verbatim nature of the participants' statements. Subsequently, the authors identified codes within the data and developed subthemes as part of the analysis process.

Thematic analysis was employed in this study, which provides a comprehensive and qualitative exploration of the data [26]. This approach allows for a thorough understanding of the data in relation to the underlying theory. To ensure transparency and rigor in reporting the research findings, the authors adhered to the Consolidated Criteria for Reporting Qualitative Research Checklist [27]. The checklist serves as a guideline to effectively present the results of qualitative research.

### 2.5 Ethical considerations

Ethical approval for this study was obtained from the review committee of Mahbubur Rahman Memorial Hospital & Nursing Institute, Bangladesh, with the approval number HRM/INC/288/10/08/2022. All interviewees were provided with comprehensive information regarding their rights to withdraw from the study, the voluntary nature of their participation, and any potential risks associated with the research. To obtain their consent, participants were requested to provide online consent through a Google Form. The confidentiality and anonymity of the participants' identities were strictly maintained throughout the study [28].

### 2.6 Rigor

To ensure the trustworthiness of the data set, this study employed various measures to evaluate its credibility, dependability, confirmability, and transferability. Member verification was conducted to enhance the credibility of the initial data collection process [29]. An audit trail was established by recording interviews and thoroughly checking the data set multiple times to safeguard the dependability of the study [30]. Triangulation was employed to enhance the confirmability of the analysis. Data saturation was achieved during the data collection process to ensure the transferability of the findings. These measures were implemented to enhance the overall rigor and reliability of the study's findings.

## 3 Results

### 3.1 Participant characteristics

This study comprised a sample of 14 frontline military nurses. The age of the respondents ranged from 26 to 42 years, with a mean age of 32.2 years (SD = 0.354). The majority of participants were females, accounting for 71.43% of the sample. Among the participants, 78.57% reported being married, while 21.43% were unmarried. In terms of educational attainment, 42.86% (N = 6) held a master's degree, while 57.14% (N = 8) had completed a bachelor's degree in nursing. Regarding work experience, 64.29% (N = 9) of the respondents had at least five years of experience, while 35.71% (N = 5) had between two and four years of experience, with a mean of 8.6 years (SD = 0.35).

### 3.2 Thematic analysis

The primary objective of this study was to investigate the survival strategies that inspired frontline military nurses in their care of COVID-19-infected patients. Eight themes were identified after analyzing the collected data, representing key patterns, and recurring elements in the nurses' experiences with coping mechanisms. These themes, as depicted in Table 2, provide a comprehensive overview of the strategies and approaches utilized by military nurses to navigate the challenges and complexities of caring for COVID-19 patients. The emergence of these themes highlights the resilience, adaptability, and resourcefulness demonstrated by frontline military nurses in their crucial role during the pandemic. In addition, these findings contribute to a deeper understanding of the strategies employed by nurses in managing and providing care for COVID-19 patients, thereby informing future practices and interventions to support healthcare professionals in similar contexts.

**Table 2:** Thematic analysis.

**Themes**	**Sub-themes**	**Codes**
**Nurses' self- strategies**	Nutrition as a source of resilience	Nutritious foods, balanced diet, mental strength, challenging conditions, high workload, night duty, pregnant, frontline fighter, COVID-19 pandemic, staff shortage, nurses, unique creativity, confidence, caring, challenging circumstances, spirits, commitment, quality care, emergency, determination, confidence, avoided negative news, prayed, meditated, exercises, relieve stress, obligated to help others, allah, good deeds, life
	Motherhood and dedication in the face of workload	
	Resilience and creativity in challenging conditions	
	Dedication, confidence, and the pursuit of excellence	
	Coping strategies for emotional resilience	
**Colleagues' strategies**	Mutual support and teamwork in nursing	Colleagues, work harder, reduce workload, nurses, couldn't sleep, caring for child, night duty, exhausted, helpful, nursing activities, home-cooked food, cakes, pleasing gestures, called for assistance, emergency, stress at work, no negative behavior, came forward, help with a smile, behavior of nurses, work pressure
	Reciprocal assistance for work-life balance	
	Supportive workplace culture through acts of kindness	
	Camaraderie and unity in times of stress	
**Nurse managers' strategies**	Effective nurse manager strategies	Coronavirus-infected patients, nursing managers, skill mix, novice and experienced nurses, frustrated, hands-on care, pandemic, manager, evaluated, profoundly, improved practice, close guidance, practical abilities, workload, COVID-19-affected patients, inspiration, challenging situation, clinical setting, masterfully organized, encouraging training sessions
	Nurse manager leadership in high-pressure situations	
	Inspirational leadership of nursing managers	
	Effective organization and training by nurse managers	
**Feelings about nursing ethical values**	Commitment to patient dignity and care	Patient dignity, ensured, adequate health care, responsible, nurse, committed, front line, standard nursing care, honest, nursing responsibilities, managing COVID-19 patients, well-being of patients, feel the patients' problems, patient's condition, relationship, needs healthcare
	Dedication to frontline nursing	
	Patient-centered care and empathy	
**Employers' strategies**	Supportive workplace resources	Employer, facilities, COVID-19 patients, transportation, housing, food, salary increments, health insurance, adequate equipment, staff, training facilities, satisfied, extra allowance, stimulus, organization, rewarded nurses, fighting against COVID-19, authorities, suggestion box, healthcare workers, innovative ideas, clinical problems, improve workplace environment
	Job satisfaction and compensation	
	Recognition and incentives for COVID-19 frontline nurses	
	Promoting innovation and collaboration through suggestion box	
**Government strategies**	Government support and incentives for frontline workers	Government, allowances, frontline fighters, inspiration, rules, visitor restrictions, hospital area, transmission, coronavirus, communities, adequate equipment, hospital, COVID-19 patients, hired nurses, steps, online training, help people, corona
	Effective government policies in pandemic control	
	Government support in healthcare infrastructure	
	Government initiatives for healthcare workforce development	
**Family members' strategies**	Spousal support in times of emotional crisis	Husband, support, coping, emotional crisis, family members, inspired, fight, destructive virus, duty, corona ward, sister, cared, little boy, grateful, time for family, patients, proud, encouragement, morale, serve patients
	Family as a source of inspiration and confidence	
	Family sacrifices and support	
	Family pride and encouragement boosting morale	
**Social people's strategies**	Community recognition and confidence boost	Organizations, community, reception awards, nurses, confidence, work, better about ourselves, duty, people, area, greeted bouquets, inspired, positive comments, community members, frontline fighter, hero, respect, society's citizens
	Community appreciation and inspiration	
	Positive community feedback and inspiration	
	Frontline heroes and community respect	

#### 3.2.1 Nurses' self-strategy

Military nurses prioritized their physical and mental well-being by consciously choosing nutritious foods. Recognizing the demanding nature of their work, nurses understood the importance of maintaining a healthy diet to sustain their energy levels and overall health. Moreover, their motivation to care for COVID-19-infected patients stemmed from a deep sense of purpose and belief in positively impacting future generations. They believed future generations would appreciate their commitment and sacrifices during the pandemic, leaving a legacy of admiration and inspiration. This underlying motivation significantly impacted their resilience and commitment to providing exceptional care amidst challenging circumstances.

I ate nutritious foods regularly to maintain my health …... eating a balanced diet helped me become mentally strong enough to deal with challenging conditions (N7, Code:107). Due to the high workload, I worked 15 days of night duty with only one night off every seven days, despite being pregnant. I just persuaded myself that one day my kid would be pleased that his mother was a frontline fighter during the COVID-19 pandemic. (N13, Code:113).

Nurses exhibited outstanding creativity, dedication, and tenacity in patient care during the COVID-19 pandemic. Their unwavering commitment to their profession and patients was evident as they faced unprecedented challenges and uncertainties. Despite their overwhelming demands, nurses continuously found innovative ways to deliver high-quality care while adapting to evolving circumstances.

Although we had a staff shortage, our nurses exhibited unique creativity and confidence in caring for infected patients. The challenging circumstances did not dampen their spirits or deter their commitment to delivering quality care (N1, Code:101). I am committed to being with the patient in an emergency. This determination makes me confident. I feel that nurses do not like to be left behind but are accustomed to being at the forefront of service (N5, Code:105). I was interested in working on a new disease (N7, Code:107).

Military nurses consciously tried to steer clear of negativity to alleviate the stress and emotional toll of their demanding work. However, they recognized the detrimental effects of dwelling on negative thoughts and emotions, so they actively sought positive perspectives and maintained an optimistic mindset. As a result, military nurses created a positive atmosphere that uplifted their spirits and motivated them to continue their vital work. In addition, religious convictions served as a profound source of inspiration for nurses serving COVID-19-infected patients. Their faith provided them with a sense of purpose and a deep-rooted belief in the value of caring for others in times of crisis. To face the difficulties and uncertainties of the pandemic, nurses found comfort, grit, and resilience in their religious principles.

I avoided negative news about COVID-19. I prayed, meditated, and did exercises to relieve stress (N9, Code:109). I feel obligated to help others because, after I die, Allah will ask me about the good deeds I performed in this life (N14, Code:114).

#### 3.2.2 Colleagues' strategies

The nurses' constant support for colleagues while caring for COVID-19 patients was impressive. They went above and beyond to offer assistance and guidance, creating a collaborative environment where knowledge and resources were shared. This teamwork improved patient care and fostered a sense of camaraderie and unity among the nursing staff. Moreover, recognizing the challenges and stressors of their demanding roles, the nurses proactively worked together to implement strategies for reducing work-related stress. Through regular debriefing sessions, self-care practices, and open communication, they prioritized their well-being and enhanced their ability to provide exceptional care.

My colleagues tended to work harder to reduce the workload of other nurses (N11, Code:111). I remember that I couldn't sleep one night because I was caring for my child, and I had night duty the next day, so I was exhausted. My coworker on night duty with me was immensely helpful in completing my nursing activities (N2, Code:102).

In times of emergency, the nurses displayed an extraordinary level of enthusiasm and dedication in supporting their colleagues. They willingly stepped up to assist one another, going beyond their assigned responsibilities. Some nurses even took the initiative to bring food and refreshments to uplift the spirits of their fellow healthcare providers. These acts of kindness and solidarity fostered a deep sense of camaraderie and unity within the nursing staff. By nurturing this strong bond, the nurses created a supportive environment where everyone felt valued, motivated, and inspired to deliver their best care. Such gestures of encouragement truly exemplified the exceptional teamwork and compassion among the nursing team.

My colleagues sometimes bring home-cooked food and cakes to please us (N6, Code:106). When I called for assistance in an emergency when my coworkers were under a lot of stress at work, no one behaved negatively; instead, everyone came forward to help with a smile (N14, Code:114). At that time, seeing the behavior of nurses under work pressure, it seemed that we were members of the same family (N5, Code:105).

#### 3.2.3 Nurse managers’ strategies

The nurse managers demonstrated a wealth of experience and expertise in identifying nurses who may have been struggling with clinical activities. They took a proactive approach, working closely with frontline nurses to provide support and guidance. Through open communication and individualized coaching, the managers helped the nurses cope with their challenges in the problematic situation. Using skill-mixed techniques, they effectively allocated resources, ensuring the clinical environment was managed efficiently. By leveraging their experience and collaborating with the frontline staff, the nurse managers played a vital role in optimizing patient care and fostering cohesion and resilience among nurses.

To deal with coronavirus-infected patients, nursing managers used a skill mix of novice and experienced nurses (N10, Code:110). While I was frustrated with the provision of hands-on care during the pandemic, my manager evaluated me profoundly and improved my practice through close guidance (N12, Code:112). We experienced nurse managers' practical abilities at an opportune time when the workload was high due to the large number of COVID-19-affected patients admitted (N14, Code:114).

The motivation and support provided by nurse managers significantly impacted the efforts of first-line nurses in caring for COVID-19 patients. Their strong leadership and guidance ensured that the clinical environment was organized and optimized for the demanding circumstances. Nurse managers efficiently allocated resources, coordinated schedules, and implemented protocols to enhance workflow and patient care. Furthermore, the training sessions organized by the managers were engaging and informative, leaving a lasting impression on the frontline nurses. These sessions gave the nurses valuable knowledge and skills, boosting their confidence and effectiveness in handling COVID-19 cases. The dedication and proactive approach of the nurse managers played a crucial role in empowering the frontline nurses and achieving optimal outcomes in patient care.

I will never forget my managers' inspiration to deal with any challenging situation (N4, Code:104). The clinical setting for caring for COVID-19 patients was masterfully organized by nurse managers (N2, Code:102). Nursing managers arranged interesting and encouraging training sessions to deal with COVID-19 patients (N7, Code:107).

#### 3.2.4 Feelings about nursing ethical values

Military nurses consistently embraced and upheld essential nursing ethical values in the challenging task of caring for COVID-19 patients. They recognized the inherent human dignity of each patient, treating them with respect, compassion, and empathy. With unwavering commitment, they dedicated themselves to providing the highest quality care, ensuring the well-being and safety of their patients. Honesty remained a core value as they communicated openly and transparently with patients and their families, sharing information, discussing treatment options, and addressing concerns. By embodying these ethical values, the nurses not only upheld the professional standards of their practice but also created a supportive and trustworthy environment that instilled confidence and comfort in the patients they cared for.

Patient dignity can be ensured by providing adequate health care, and I am responsible for this (N3, Code:103). As a nurse, I am committed to working at the front line in any situation (N8, Code:108). I feel I need to provide standard nursing care. I have to be honest; I can't avoid any nursing responsibilities in managing COVID-19 patients (N7, Code:107).

It was evident that frontline nurses consistently demonstrated empathy and positively interacted with patients infected with the coronavirus. They approached each patient with genuine care, listening attentively to their concerns and providing emotional support during difficult times. By displaying empathy, the nurses created a comforting and reassuring environment for patients, fostering a sense of trust and understanding. These compassionate interactions played a vital role in promoting the patients' well-being and recovery, highlighting the frontline nurses' exceptional dedication and professionalism.

For the well-being of the patients, I must feel the patients' problems whatever the patient's condition (N11, Code:111). As a nurse, my relationship should be with someone who needs healthcare (N1, Code:101).

#### 3.2.5 Employers’ strategies

The employer played an essential role in supporting the healthcare organization during the challenging circumstances of the COVID-19 pandemic. They ensured that the necessary resources, such as personal protective equipment (PPE) and medical supplies, were readily available to the employees. Additionally, the employer provided sufficient facilities to accommodate the increased demand for patient care, creating a conducive environment for healthcare professionals. This proactive approach by the employer helped to alleviate concerns and boost employee satisfaction, enabling the healthcare organization to navigate the unique challenges presented by the pandemic effectively.

The employer has given us enough facilities to take care of COVID-19 patients. Such as transportation, housing, food, good salary increments, and health insurance (N3, Code:103). We have adequate equipment, staff, and training facilities to manage patients (N1, Code:101). I was satisfied with my salary for COVID-19 patient care (N8, Code:108).

Employers recognized the invaluable efforts of frontline nurses and incentivized their clinical work by offering additional financial compensation and rewards. These measures aimed to acknowledge and appreciate their dedication, serving as a motivating factor for the nurses to continue providing exceptional care to COVID-19 patients.

We received an extra allowance for working with COVID-19 patients, which served as a stimulus (N10, Code:110). Our organization has rewarded nurses for fighting against COVID-19 (N6, Code:106).

Healthcare employers proactively sought feedback from healthcare workers to gain insights into clinical issues and improve the quality of services. They implemented creative methods such as anonymous surveys, suggestion boxes, and regular meetings to encourage open communication. This collaborative approach allowed employers to address concerns, make necessary improvements, and ensure that the healthcare organization continuously evolved to meet the needs of both patients and staff.

The authorities have set up a suggestion box. In this activity, healthcare workers write down their innovative ideas to solve clinical problems and improve the workplace environment (N13, Code:113).

#### 3.2.6 Government strategies

Governments recognized the critical role of nurses in caring for COVID-19 patients and provided financial support as an incentive for their involvement. Additionally, governments imposed mandatory rules and regulations on healthcare organizations to enhance clinical settings and ensure suitable working environments for healthcare staff. These measures helped prioritize healthcare professionals' well-being and create optimal conditions for them to deliver quality care in the fight against the pandemic.

The government gave us allowances as frontline fighters to inspire us (N9, Code:109). Government rules on visitor restrictions in the hospital area considerably helped to prevent the transmission of coronavirus to communities (N4, Code:104).

The government's initiative proved highly advantageous by ensuring the availability of essential resources. Governments timely hired additional military nurses, effectively reducing the workload on existing healthcare professionals. Furthermore, nurses greatly appreciated the government's provision of online training programs focused on COVID-19-infected patient management. These training sessions equipped nurses with the knowledge and skills needed to provide optimal care, reinforce their confidence, and enhance their ability to navigate the challenges posed by the pandemic.

The government provided adequate equipment to our hospital to handle COVID-19 patients (N12, Code:112). At that moment, the government hired nurses in a few steps (N2, Code:102). We learned a lot from the government's online training about how to help people with Corona (N8, Code:108).

#### 3.2.7 Family members' strategies

Military nurses drew inspiration from their husbands and family members, who played a pivotal role in helping them adapt to the challenges of the pandemic situation. Their support, understanding, and encouragement provided nurses with the emotional strength and resilience needed to navigate the demanding circumstances. Husbands and family members served as pillars of support, reminding nurses of their importance, and reminding them of the impact of their work, fueling their dedication and commitment to caring for COVID-19 patients.

My husband gave me a lot of support to cope with the emotional crisis (N4, Code:104). My family members have always inspired me to fight the destructive virus confidently (N10, Code:110).

Military nurses are immensely grateful to their family members for their support and encouragement. The backing from their loved ones significantly boosted nurses' morale in their tireless fight against the COVID-19 pandemic. Family members became a source of strength and motivation, reminding nurses of the importance of their work and the positive impact they were making. This support strategy was critical in sustaining nurses' resilience and dedication, allowing them to persevere in providing care and saving lives.

Because of my duty at Corona Ward, I came home every 21 days while my sister cared for my little boy. I am grateful to her for giving me time for my family. I was able to take care of patients for her support (N5, Code:105). My family is proud of me and has given me a lot of encouragement. This has increased my morale to serve patients (N14, Code:114).

#### 3.2.8 Social people's strategies

Numerous community-based social organizations recognized and rewarded military nurses for their unwavering dedication to caring for coronavirus-infected patients. The appreciation and respect from the public significantly reinforced military nurses' confidence, motivating them to engage more confidently in patient care. These recognition initiatives served as a source of inspiration and motivation for military nurses and highlighted the invaluable contribution of healthcare professionals in the fight against the pandemic. Such support from the community fostered a strong sense of pride and motivation among nurses.

Several organizations in our community have given reception awards to some of our nurses, which has made us more confident at work and made us feel better about ourselves (N9, Code:109). On my way home from duty, people in my area often greeted me with bouquets, which inspired me greatly (N8, Code:108).

The positive sentiments and acceptance of community members profoundly impacted nurses' willingness to participate in COVID-19 patient care. The outpouring of support and appreciation from the community created a sense of purpose and motivation among nurses. Knowing their efforts were valued and recognized, nurses felt a strong sense of duty to step forward and provide care to those in need. The community's positive influence inspired and empowered nurses to face the challenges of the pandemic with resilience and compassion.

I was even more inspired when I heard positive comments from community members (N11, Code:111). As a frontline fighter, I felt like a hero because of the respect I gained from society's citizens (N3, Code:103).

## 4 Discussion

Military nurses employed various self-strategy measures to combat COVID-19 infections during the pandemic [31]. Healthcare professionals adopt distinct self-strategies to enhance their abilities and competencies [32]. The utilization of self-management strategies enables nurses to engage in appropriate nursing care, reducing work stress and job dissatisfaction [33]. These self-strategies considerably impact nurses' willingness to participate in clinical settings and contribute to positive healthcare outcomes [34].

Nurses found inspiration to work in challenging circumstances during this pandemic through the strategies of their colleagues. Nursing is inherently a team-oriented profession [35]. Effective interpersonal communication among nursing colleagues plays a pivotal role in achieving favorable patient outcomes [36]. Nurses' perceptions of support from their colleagues aid them in delivering better care and coping with job-related stress [37]. The support received from colleagues has a positive influence on job satisfaction [38].

This study unveiled that nurse managers' strategies were centered on motivating frontline nurses to engage in the care of coronavirus-affected patients within a challenging working environment. Nurse managers hold a pivotal role in fostering nursing skills development [39] and serve as a wellspring of motivation for bedside nurses [40]. Nurse managers guide nurses in cultivating problem-solving abilities, honing decision-making skills, and enhancing critical thinking capabilities [41,42]. Nurse managers' approaches can incentivize nurses to collaborate as a team during high-stress situations [43] and wield a significant influence in enhancing overall organizational performance [44].

Nurses were further encouraged to remain committed to COVID-19 critical patients owing to their unwavering commitment to professional and ethical values. Nurses are entrusted with upholding a spectrum of ethical ideals encompassing humanity, precision in caregiving, autonomous judgment, social equity, accountability, humane interactions, professional and personal proficiency, trustworthiness, and empathy [45]. Ethical values serve as compasses for healthcare workers in making sound decisions and judicious judgments [46]. Furthermore, employer strategies played a pivotal role in aiding frontline nurses in navigating the challenges posed by the COVID-19 pandemic. Employers are responsible for adapting the healthcare system, navigating health insurance policies, and structuring employees' health benefits [47]. Effective employer strategies contribute to improved service delivery and employee satisfaction [48]. Nursing retention hinges on the implementation of effective employer strategies [49], while the absence of such techniques leads to job dissatisfaction [6,50].

Throughout the pandemic, the government implemented strategies to encourage nurses' involvement in inpatient care. Government planning, policymaking, and developmental strategies contribute significantly to the provision of appropriate healthcare services [51]. Government regulations play a critical role in governing healthcare settings [52], and the quality of services is contingent on how effectively the government provides guidance to organizations [53]. Government strategies address the demands of both employers and employees [54]. Furthermore, frontline nurses and their families demonstrated unwavering dedication during this pandemic. Supportive strategies employed by family members influence nurses' decisions when dealing with COVID-19 patients [55]. Family support assists employees in maintaining a healthy work-life balance [56]. Effective family management and workplace involvement are mutually beneficial [57].

Social support also motivated frontline nurses caring for COVID-19-infected patients. Love, respect, and recognition from social circles motivate frontline nurses to work in inpatient care. However, negative societal perceptions can reduce an employee's interest in their employment [58]. Overall, the adoption of self-strategies, support from colleagues, guidance from nurse managers, adherence to ethical values, employer strategies, government initiatives, and family and social support play crucial roles in motivating and empowering frontline nurses in their efforts to provide quality care during the challenging times of the COVID-19 pandemic. Understanding and nurturing these factors are essential for sustaining the resilience and dedication of healthcare professionals in the face of unprecedented healthcare crises.

## 5 Implications of this study

The study's findings carried significant implications for healthcare systems, particularly in responding to infectious disease outbreaks like COVID-19. Policymakers must recognize the critical role played by military nurses in pandemic response, emphasizing the necessity of investing in their training, support, and overall well-being. This involves providing specialized training in psychological resilience and coping strategies to help nurses manage the emotional toll of their work. To address unique emotional challenges faced during pandemics, comprehensive support systems, including robust mental health and counseling services, should be prioritized, with a focus on strategies to alleviate work-related stress and burnout.

Furthermore, this study highlighted the importance of ethical values in nursing practice during crises, calling for the incorporation of ethical training and guidelines into nursing education and practice. This integration is crucial for guiding nurses through difficult ethical decisions and ensuring patient-centered care. Healthcare organizations should devise contingency plans that consider the specific needs of military nurses, including flexible scheduling, access to childcare support, and resources for remote work.

The implications extend to nursing management practices, emphasizing the prioritization of staff well-being. Drawing insights from the diverse survival strategies employed by military nurses, nursing managers should implement tailored support mechanisms, recognizing and addressing the emotional toll on nurses caring for infected patients. Measures such as regular check-ins, access to counseling services, and fostering a supportive work environment are crucial. Encouraging a culture of collaboration and peer support among nurses, facilitated through peer support programs, team-building activities, and forums for sharing experiences, is highlighted.

Additionally, nurse managers should be well-versed in ethical challenges, understanding the impact of nursing ethical values on decision-making. This understanding enables nursing leaders to guide their staff through difficult ethical dilemmas [59], fostering a culture of ethical practice. Advocacy for resources and support from healthcare institutions and government bodies, including adequate staffing levels, access to personal protective equipment, and policies prioritizing nurse safety and well-being, is essential for effective nursing management.

To advance understanding, a quantitative study is recommended to evaluate the statistical significance of variables associated with survival mechanisms and care quality. Furthermore, a strong recommendation is made for a study to develop a survival technique scale, providing insight into the level of nurses' survival abilities. These research avenues are crucial for enhancing the resilience and effectiveness of frontline nurses in critical healthcare situations.

## 6 Limitations of this study

While this study provides valuable insights into the survival strategies of military nurses caring for COVID-19 patients in Bangladesh, several limitations should be acknowledged. Firstly, the study's reliance on purposive sampling might introduce selection bias. The participants were selected based on specific criteria, including a minimum of two years of experience, employment in larger COVID-19 facilities, and age criteria. This could potentially exclude the perspectives of less experienced nurses or those working in smaller healthcare settings, limiting the generalizability of the findings to a broader population of military nurses. Secondly, the study's data collection process primarily relied on online video interviews conducted in the Bangla language. While this approach was necessary to accommodate participants' preferences and ensure their comfort, it might have introduced language and cultural nuances that could affect the accuracy of translation and interpretation. Despite the involvement of a data translation specialist, some subtleties in participants' experiences and expressions may have been lost in translation.

In addition, the research was conducted during a specific time frame, from January to February 2023, which captures a snapshot of military nurses' experiences during the COVID-19 pandemic. The evolving nature of the pandemic and the healthcare system's response to it means that the survival strategies employed by nurses may have evolved over time. Thus, the findings might not fully represent the experiences of military nurses in later stages of the pandemic or in different healthcare contexts. Furthermore, the study's qualitative nature emphasizes depth over breadth. While it provides rich insights into the survival strategies of a limited sample of military nurses, it may not capture the full spectrum of strategies employed by nurses in different regions or healthcare settings. Therefore, caution should be exercised when attempting to generalize these findings beyond the specific context of this study. Lastly, as with any qualitative research, there is the potential for researcher bias during data analysis and interpretation. Despite efforts to maintain rigor and transparency, the researchers' perspectives and preconceptions could have influenced the identification and characterization of themes.

## 7 Conclusions

The utilization of effective survival strategies dramatically influences the ability of nurses to cope with complex clinical situations. These mechanisms mentally motivate nurses to overcome challenging circumstances, manage stress at work, and cultivate a positive interest in their profession, especially when dealing with critical patients. Developing nurses' survival mechanisms is paramount for enhancing the overall quality of care and inspiring them to persist in their respective fields. By strengthening their survival skills, nurses are better equipped to cope with work demands, ultimately leading to improved patient outcomes and a more resilient healthcare workforce.

## Funding information

There was no external fund taken for this current research.

## Declaration of interest's statement

The authors declare no conflict of interest.

## Data availability statement

The datasets used and/or analyzed during the current study are available from the corresponding author on reasonable request.

## CRediT authorship contribution statement

**Mst Rina Parvin:** Writing – review & editing, Writing – original draft, Visualization, Validation, Software, Resources, Project administration, Methodology, Investigation, Formal analysis, Data curation, Conceptualization. **Priyanka Das Sharmi:** Writing – review & editing, Visualization, Resources, Methodology, Investigation, Data curation. **Ibne Kayesh:** Writing – review & editing, Visualization, Resources, Methodology, Investigation, Data curation. **Moustaq Karim Khan Rony:** Writing – review & editing, Writing – original draft, Visualization, Validation, Supervision, Software, Resources, Project administration, Methodology, Investigation, Formal analysis, Data curation, Conceptualization.

## Declaration of competing interest

The authors declare that they have no known competing financial interests or personal relationships that could have appeared to influence the work reported in this paper.
